# Supplementary material for: STK16 promoted colorectal cancer progress in a c-MYC signaling-dependent manner
Source: Mol Med. 2024 Apr 15;30:50. doi: 10.1186/s10020-024-00816-9 (PMC11020453; doi:10.1186/s10020-024-00816-9)
Supplement: Supplementary file 5 — Supplementary Material 5 [file 10020_2024_816_MOESM5_ESM.docx]

**Supplementary Figure 1. STK16 had a Positive Correlation with Cancer Patients’ Overall Survival Time**. **A**. Analyzing the expression level of STK16 in tumor tissues and matched normal tissues using TCGA database. **B**. Drawing Kaplan-Meier Plot in various cancers using TCGA database, patients grouped by the expression level of STK16.

**Supplementary Figure 2. STK16 Positively Regulated c-MYC Signaling**. **A**. GSEA analysis of GEO datasets, patients grouped by the expression level of STK16. **B, C**. IB assays to assess the expression of c-MYC signaling-related proteins in cancer cells stably expressing vector, STK16 WT, or STK16 T198A. **D, E**. IB assays to assess the expression of c-MYC signaling-related proteins in cancer cells treated with DMSO or STK16-IN-1 (5uM, 10uM). **F, G**. RT-PCR assays to detect the mRNA expression level of c-MYC in identified cells. **H**. Representative IHC images of colorectal cancer tissues. All IB assays were conducted three times, and consistent results were obtained. Statistical analysis was conducted using Student's t-test.

**Supplementary Figure 3. STK16 Phosphorylated c-MYC**. **A**. IB and IP assays confirmed the binding of STK16 and c-MYC. **B**. IB and IP assays to assess the phosphorylation level of c-MYC in RKO cells stably expressing vector or STK16 WT. **C**. IB and IP assays to assess the phosphorylation level of c-MYC in RKO cells treated with DMSO or STK16-IN-1. **D**. Treating cells with MG132 and IB assays to assess the expression level of c-MYC in cancer cells stably expressing c-MYC WT, c-MYC S452A, or c-MYC S452E.
