# Supplementary material for: STK16 promoted colorectal cancer progress in a c-MYC signaling-dependent manner
Source: Mol Med. 2024 Apr 15;30:50. doi: 10.1186/s10020-024-00816-9 (PMC11020453; doi:10.1186/s10020-024-00816-9)
Supplement: Supplementary file 6 — Supplementary Material 6 [file 10020_2024_816_MOESM6_ESM.docx]

**STK16 promoted colorectal cancer progress in a c-MYC signaling-dependent manner**

Li Peng, Liu Guangshi, Lai Bijiang· Wusman, Li Tao*

Author affiliation: Gastrointestinal Surgery department, People’s Hospital of Xinjiang Uygur Autonomous Region, Urumqi City, Xinjiang Province, China

*Corresponding author: Li Tao

Corresponding E-mail: lp20014@163.com

**Abstract**

**Background**

Colorectal cancer standed as a global health challenge, ranking third in cancer incidence and second in cancer-related deaths worldwide. A deeper understanding of the intricate mechanisms driving colorectal cancer development was pressing need. STK16 had garnered attention in recent researches, while its involvement in cancer had been minimally explored. c-MYC had emerged as a key player in cancer biology. Due to its complex structure, multifunctionality, and intricate interactions, directly inhibiting the activity of c-MYC proves to be challenging. Hence, current research was directing efforts towards modulating c-MYC expression levels.

**Methods**

Immunoblot, Immunohistochemistry and immunoprecipitation assays were conducted to assess the indicated protein expression levels. RT-PCR was performed to detect the corresponding mRNA expression levels. The proliferation, migration, invasion, and colony formation abilities of the specified cancer cells were investigated using CCK8 assays, Brdu assays, transwell assays, and colony formation assays, respectively. Cellular and animal experiments were performed to investigate the correlation between STK16 signaling and c-MYC signaling.

**Results**

STK16 plays a positive regulatory role in the progression of colorectal cancer. Delving into the molecular mechanisms, we unveiled that STK16 phosphorylated c-MYC at serine 452, a pivotal event hindering the ubiquitin-proteasome pathway degradation of c-MYC. Importantly, colorectal cancer proliferation mediated by STK16 was found to be dependent on the phosphorylation of c-MYC at S452. Furthermore, the researchers demonstrated that STK16 knockout or pharmacological inhibition significantly curtailed colorectal cancer proliferation and c-MYC expression in in vivo animal models.

**Conclusion**

We discovered that STK16 phosphorylates c-MYC at serine 452, hindering its degradation via the ubiquitin-proteasome pathway. STK16 inhibition, either genetically or pharmacologically, effectively curtails cancer growth and c-MYC expression in vivo. These findings highlight STK16 as a potential therapeutic target for colorectal cancer.

**Introduction**

Colorectal cancer was the third most common cancer and the second leading cause of cancer-related deaths globally.(1, 2) Although there were multiple treatment options for colon cancer, such as chemotherapy, radiotherapy, targeted therapy, immunotherapy, and surgery, tumor recurrence appeared to be inevitable for some patients, especially those in advanced stages.(3, 4) Therefore, gaining a deeper understanding of the potential mechanisms underlying the development of colorectal cancer was crucial for identifying new targeted treatment strategies.

STK16 was a serine/threonine protein kinase with a complete kinase catalytic domain, a very short N-terminal domain, and a brief C-terminus.(5) STK16 was involved in various cellular signaling pathways and regulated multiple cellular processes, including the TGF-β signaling pathway activation, TGN (Trans-Golgi Network) protein secretion and sorting, as well as Golgi apparatus assembly and cell cycle arrest.(5-10) However, limited research had been conducted on the role of STK16 in cancer. One study demonstrated that STK16 induces VEGFA expression, promoting fibrosarcoma growth, while another investigation established that the loss of STK16 inhibited lung cancer tumor growth by suppressing AKT signaling.(11, 12) Additionally, there was no research available on the role of STK16 in colorectal cancer.

c-MYC (the protein encoded by the MYC gene) was a transcription factor and a crucial regulator of cell cycle control and cellular proliferation.(13) In normal cells, c-MYC was involved in regulating various biological processes, including cell growth, differentiation, metabolism, and apoptosis.(14) However, when the regulation of c-MYC was disrupted, it might play a significant role in cancer. It had been widely reported that the overexpression of c-MYC was closely associated with rapid proliferation, apoptosis inhibition, promotion of angiogenesis, metabolic reprogramming, invasion, and metastasis in cancer cells.(15) Hence, c-MYC was considered a potential therapeutic target. However, pharmacological intervention to inhibit c-MYC faced multiple challenges. The complex structure and multi-functionality of c-MYC, as well as its interactions with multiple elements in biological networks, made it difficult to find specific targets. Moreover, the short half-life of c-MYC, and lack of typical enzyme active sites all increased the difficulty in developing drugs to inhibit it.(16-18) Current research focused on finding more effective inhibition strategies by modulating its expression levels.

Here, we had demonstrated for the first time that STK16 functioned as an oncogene in colorectal cancer. Its expression level exhibited a positive correlation with the proliferation, migration and invasion ability of colorectal cancer. Mechanistically, we had confirmed that STK16 phosphorylated c-MYC at serine 452, and c-MYC S452 phosphorylation was a key event that impeded the ubiquitin-proteasome pathway degradation of c-MYC. Besides, the proliferation of colorectal cancer mediated by STK16 depended on the phosphorylation of c-MYC at S452. Furthermore, we had revealed that STK16 knock-out or the pharmacological inhibition of STK16 significantly inhibited colorectal cancer proliferation and c-MYC expression in vivo.

**Material and methods**

**Cell lines**

RKO, Lovo, and HEK 293T cell lines were obtained from the American Type Culture Collection. Dulbecco's Modified Eagle Medium (DMEM, Thermo Fisher Scientific) was employed for cell culture. The medium was supplemented with 10% (v/v) fetal bovine serum (Gibco) and Penicillin-Streptomycin Mix (MedChemExpress). All cell lines were maintained in a 5% CO2 atmosphere at 37°C.

**Agents**

Commercially available antibodies for STK16 (ab228608, Abcam), GAPDH (A19056, ABclonal), c-MYC (18583, Cell Signaling Technology), GLUT1 (73015, Cell Signaling Technology), β-actin (AC038, ABclonal), Flag tag (AE005, ABclonal), CDK4 (ab108357, Abcam), and HA tag (ab9110, Abcam) were used. MG132 (HY-13259) and STK16-IN-1 (HY-101270) were purchased from MedChemExpress.

**Cell lines construction**

Silencing lentivirus was generated using pLKO.1-shRNAs and the MD2-G, PPAX plasmids in a three-package system. pLKO.1-AS3W was used for indicating genes. MD2-G and PPAX plasmids in a three-package system were employed to produce the overexpression lentivirus. The sequences of shc-MYC were as follows: shc-MYC#1: 5’- CCATAATGTAAACTGCCTCAA-3’; shc-MYC#2: 5’- CAGTTGAAACACAAACTTGAA-3’; shc-MYC#3: 5’- CCTGAGACAGATCAGCAACAA-3’. Puromycin was utilized to screen infected cells until all control cells died. For the construction of STK16 and c-MYC knock-out cell lines, sgRNAs were cloned into pSpCas9(BB)-2A-Puro (PX459) V2.0 plasmids. These plasmids were then transfected into RKO and Lovo cells. After 48 hours, puromycin was applied to screen the infected cells until all control cells died. The remaining cells were seeded into a 96-well plate for monoclonal screening. The sgRNAs for STK16 and c-MYC were as follows: sg c-MYC #1: 5’-GACGGACAGGATGTATGCTG-3’; sg c-MYC #2: 5’-GCTCCTCTGCTTGGACGGAC-3’; sg c-MYC #3: 5’-GGATAGTCCTTCCGAGTGGA-3’; sgSTK16#1: 5’-GAGAGCAGACACACAGCGCG-3’; sgSTK16#2: 5’-GAGATGCGCTGGGGGCCCAG-3’; sgSTK16#3: 5’-GCTCTTCCAGGAGATGCGCT-3’. Western blots and RT-PCR assays were applied to validate the successful construction.

**Immunohistochemistry (IHC)**

Two independent professors assessed the results of an immunohistochemistry (IHC) experiment. The IHC score was determined using the IRS system, where the percentage score indicated the proportion of cells expressing the target protein in a specific tissue area: 1 (<10%), 2 (10–50%), 3 (50–75%), and 4 (>75%). The intensity score was categorized as strong positive (3), moderate positive (2), weak positive (1), or negative (0). The ultimate IHC score was calculated by multiplying the percentage score by the intensity score.

**BrdU assay**

The pretreated cells were plated in a 24-well plate. Cell proliferation ability was assessed using the BeyoClick™ EdU Cell Proliferation Kit with Alexa Fluor 555 (C0075L, Beyotime Biotechnology) following the instructions provided.

**CCK8 assay**

CCK-8 assays were performed using the Cell Counting Kit-8 (RM02823, ABclonal) in accordance with the provided instructions.

**Colony formation assays**

Pretreated cells, ranging from 100 to 300, were seeded into a 6-well plate. The medium was changed every three days. After two weeks, colonies were fixed with 4% paraformaldehyde and stained with crystal violet.

**Trans-well assays**

Cells, ranging from 1 to 3 × 10^4, were seeded with 300μl of serum-free medium into the upper chamber, while 400μl of complete medium was added to the lower chamber. Mitomycin (1 µg/mL) was used to inhibit cancer cell proliferation. After 12-16 hours, cells were fixed with 4% paraformaldehyde and stained with crystal violet. Cells on the inner membrane were removed using a cotton swab, and cell images were captured using inverted microscopes.

**Western blot assays**

The pretreated cells were lysed with NP-40 lysis buffer. Protein concentration was measured using the BCA Protein Assay Kit (P0012, Beyotime Biotechnology) following the provided instructions. Proteins were separated through electrophoresis in pre-made sodium dodecyl sulfate-polyacrylamide minigels (Tris-HCL SDS-PAGE), then transferred onto a PVDF membrane. The membrane was incubated with primary antibodies overnight at 4°C and then with HRP-conjugated secondary antibodies for 2 hours at room temperature. Protein signaling was detected using a chemiluminescent solution.

**Immunoprecipitation assays**

Colorectal cancer samples were acquired from the People’s Hospital of Xinjiang Uygur Autonomous Region. Primary antibodies were initially incubated with Protein L Magnetic Beads (HY-K0205; MCE) for 2 hours at room temperature, and subsequently, cell lysis was combined with pre-treated beads overnight at 4°C. The beads were washed three times using PBS and denatured for 10 minutes at 100°C, followed by western blots.

**Animal assays**

Nude female mice, aged 6-8 weeks, were procured from Beijing Huafukang Bioscience Company. 3-4 × 10^6 pretreated cells in 100μl PBS were injected into the backs of the mice. STK16-IN-1 (10mg/kg) was intraperitoneally injected once a day. Tumor volume was measured at the specified times. As for the survival time, the humane endpoints were used according to Chinese Guidelines Of Assessment For Humane Endpoints In Animal Experiments (RBT 173-2018). After the designated time, mice were sacrificed, and tumors were isolated, weighed, and fixed with 4% paraformaldehyde.

**Databases**

TCGA datasets for colon cancer and rectal cancer were obtained from the TCGA database (https://xenabrowser.net/datapages/). Datasets for GSE31595, GSE24549, GSE36864, GSE63216, and GSE24549 were downloaded from the PubMed database (https://www.ncbi.nlm.nih.gov/pubmed/). GSEA analysis was conducted using GSEA software.

**Statistical analysis**

Statistical analysis was performed using SPSS 20.0 and GraphPad Prism 9 software. Differences were assessed using Student's t-test for two groups or ANOVA for multiple groups. The Chi-Square or Pearson's correlation test was employed to examine the correlation between variables. A p-value less than 0.05 was considered statistically significant.

**Results**

**STK16 was found to be upregulated in colorectal cancer**

To evaluate its role, we initially examined STK16 expression in colorectal cancer and normal colorectal tissue using TCGA databases. The results revealed a significant overexpression of STK16 in colorectal cancer compared to normal colorectal tissues (Figure 1A-B). Subsequently, 31 pairs of colorectal cancer tissues and adjacent normal tissues were collected. IHC analysis demonstrated a substantial upregulation of STK16 in colorectal cancer (Figure 1C-E), which was further validated by western blot results (Figure 1F). Furthermore, an additional set of 63 colorectal cancer samples was subjected to IHC assays. The outcomes indicated that patients with higher T stage and clinical stage exhibited elevated STK16 expression levels (Figure 1G-I). Additionally, patients were stratified based on STK16 expression, and Kaplan-Meier analysis revealed that those with higher STK16 expression had poorer clinical overall survival times (Figure 1J). Similar findings were observed in Kaplan-Meier analysis for TCGA database of colorectal cancer (Figure 1K). Additionally, the role of STK16 in other types of cancers was explored. The results demonstrated overexpression of STK16, and patients with higher STK16 expression levels had worse clinical prognoses in most types of cancer (Figure S1). Overall, these results suggest that STK16 may function as an oncogene in the progression of colorectal cancer.

**STK16 overexpression was observed to promote the proliferation and metastasis of colorectal cancer**

To investigate its role further, we utilized lentivirus to establish RKO and Lovo cells with stable ectopic expression of STK16. Western blots confirmed the successful construction of these cells (Figure 2A). CCK8 and BrdU assays were conducted to evaluate whether the overexpression of STK16 affected colorectal cancer cell proliferation. The results indicated that cells with stable ectopic expression of STK16 exhibited enhanced proliferation ability (Figure 2B-D). Considering the inevitability of distant metastasis in the progression of colorectal cancer, we also examined the impact of STK16 on cell colony formation and metastatic potential. Colony formation assays revealed that cancer cells with stable ectopic expression of STK16 displayed a more robust ability for colony formation (Figure 2E-F). Furthermore, these cells exhibited higher migration and invasion abilities compared to control cancer cells (Figure 2G-H). In summary, these findings confirm that the overexpression of STK16 significantly enhances the proliferation and metastatic capabilities of cancer cells.

**Loss of STK16 inhibited colorectal cancer cell proliferation and metastasis**

To further affirm the biological function of STK16 in colorectal cancer proliferation and metastasis, we employed the Crisp-cas9 system to knock out STK16 in RKO and Lovo cancer cells (Figure 3A). CCK8 assays confirmed that the loss of STK16 significantly hindered colorectal cancer cell proliferation (Figure 3B). This finding was further supported by BrdU assays (Figure 3C-D). Moreover, we demonstrated that STK16 knock-out markedly suppressed the colony formation, migration, and invasion abilities of cancer cells (Figure 3E-H). In summary, these results provide evidence that STK16 plays a positive regulatory role in cancer cell proliferation and metastasis.

**STK16 positively regulated MYC signaling by stabilizing MYC**

We demonstrated that STK16 may function as an oncogene in colorectal cancer, positively correlating with cancer cell proliferation and metastasis. However, the detailed mechanism underlying STK16's regulation of colorectal cancer progression remained unclear. To address this, we analyzed TCGA and GEO databases. GSEA analysis for TCGA database indicated that STK16 positively activated the MYC signaling pathway in both colon and rectal cancers (Figure 4A). Similar results were observed in various GEO datasets (Figure S2A). We then examined the expression levels of c-MYC and its downstream genes (GLUT1 and CDK4) in colorectal cancer cells ectopically expressing STK16 or sgSTK16. Results revealed that STK16 overexpression significantly upregulated the expression of c-MYC, GLUT1, and CDK4, while silencing STK16 notably suppressed their expression levels (Figure 4B and Figure S2B). Additionally, we demonstrated that STK16 WT, but not the enzyme-deficient mutant STK16 T198A, could upregulate c-MYC, GLUT1, and CDK4 expression.(19) Treatment with the STK16 inhibitor, STK16-IN-1, decreased c-MYC and GLUT1 expression (Figure S2C-E).(20) These findings indicated that STK16-mediated c-MYC expression depended on the enzyme activation of STK16. Interestingly, STK16 had no effect on the mRNA expression of c-MYC (Figure S2F-G), suggesting that STK16 might post-translationally regulate c-MYC expression. To explore potential post-translational regulation mechanisms, we considered autophagy-lysosome and ubiquitin-proteasome pathways. Inhibiting the autophagy-lysosome pathway with chloroquine (CQ) did not affect STK16-mediated c-MYC upregulation, while inhibiting the ubiquitin-proteasome pathway with MG132 abolished this effect (Figure 4C). Further experiments using his-ubiquitin plasmids and MG132 showed that STK16 overexpression reduced the polyubiquitination level of c-MYC, suggesting that STK16 mediates c-MYC upregulation via the ubiquitin-proteasome pathway (Figure 4D). Consistent results were observed in loss-of-function experiments (Figure 4E-F). We also assessed the degradation rate of c-MYC using cycloheximide (CHX) to inhibit protein synthesis. Results showed that STK16 WT, but not STK16 T198A, significantly reduced the degradation rate of c-MYC (Figure 4G-H). Moreover, IHC assays in colorectal cancer tissues demonstrated a positive correlation between STK16 and c-MYC expression levels (Figure 4I and Figure S2H). In summary, these results suggest that STK16 activates the MYC signaling pathway by preventing the polyubiquitination of c-MYC.

**STK16 stabilized c-MYC by phosphorylating c-MYC at serine 452**

We demonstrated that STK16 post-translationally regulates c-MYC expression, and the kinase activity of STK16 is crucial for this process. Consequently, we hypothesized that STK16 might phosphorylate c-MYC. To test this hypothesis, we initially conducted co-immunoprecipitation assays to examine the binding between STK16 and c-MYC. Significantly, we observed binding between STK16 and c-MYC in RKO and Lovo cells (Figure 5A and Figure S3A). We then used a pan-serine/threonine phosphorylation antibody to assess the phosphorylation level of c-MYC in various cancer cells. Results showed that the gain of STK16, but not STK16 T198A, upregulated the phosphorylation level of c-MYC (Figure 5B and Figure S3B). Conversely, both loss of STK16 and the pharmacological inhibitor (STK16-IN-1) significantly decreased the phosphorylation level of c-MYC (Figure 5C and Figure S3C). Next, we sought to identify the serine/threonine site on c-MYC that could be phosphorylated by STK16. Co-immunoprecipitation assays using c-MYC deletion mutants revealed that the deletion of amino acids 422-454 abolished the interaction between c-MYC and STK16 (Figure 5D). Further experiments with additional c-MYC deletion mutants narrowed down the binding region to amino acids 422-454, specifically amino acids 422-421 (Figure 5E). Sequence analysis indicated two serine sites (S430 and S452) within the 422-454 amino acid region of c-MYC. Mutant plasmids were constructed for these serine sites (S430A and S452A, phospho-deficient mutants). Co-transfection experiments demonstrated that STK16 overexpression increased the phosphorylation level of c-MYC S430A mutant but not c-MYC S452A mutant (Figure 5F). These results established that STK16 phosphorylates c-MYC at serine 452. Subsequently, we generated RKO and Lovo cells ectopically expressing c-MYC WT, c-MYC S452A, or c-MYC S452E (phospho-mimetic mutants) using the Crispr-Cas9 system (Figure S3D). Immunoprecipitation assays showed that the c-MYC S452A mutant had a higher polyubiquitin level than c-MYC WT, while the c-MYC S452E mutant had a lower polyubiquitin level than c-MYC WT (Figure 5G). Consistently, cells expressing c-MYC S452A had lower expression levels of GLUT1 and CDK4 compared to cells expressing c-MYC WT, whereas cells expressing c-MYC S452E had higher expression levels of GLUT1 and CDK4 compared to cells expressing c-MYC WT (Figure 5H). Furthermore, proliferation assays indicated that cells expressing c-MYC S452A had lower proliferation ability than cells expressing c-MYC WT, while cells expressing c-MYC S452E had higher proliferation ability than cells expressing c-MYC WT (Figure 5I-K). Overall, these findings demonstrate that STK16 phosphorylates c-MYC at serine 452, and c-MYC S452 phosphorylation is essential for its stability.

**STK16-mediated cancer cell proliferation relied on c-MYC S452 phosphorylation**

To further investigate the relationship between STK16 and c-MYC, we generated four types of colorectal cancer cells stably expressing vector+shnc, STK16+shnc, STK16+shc-MYC#1, STK16+shc-MYC#2 using lentivirus. Western blot results demonstrated that STK16 overexpression upregulated the expression of GLUT1 and CDK4, an effect abrogated by c-MYC silencing (Figure 6A). Next, we assessed the proliferation ability of these four types of cancer cells through CCK8 and BrdU assays. The results indicated that the gain of STK16 enhanced the proliferation ability of cancer cells, while c-MYC silencing counteracted this effect (Figure 6B-D). Furthermore, we generated four types of colorectal cancer cells stably expressing vector+c-MYC WT, STK16+c-MYC WT, vector+c-MYC S452A, and STK16+c-MYC S452A using lentivirus and the Crispr-Cas9 system. Downstream gene expression levels of the c-MYC signaling pathway in these cells were assessed by western blot. Results showed that STK16 overexpression upregulated the expression levels of c-MYC, GLUT1, and CDK4 in c-MYC WT cancer cells, but not in c-MYC S452A cancer cells (Figure 6E). Consistently, STK16 overexpression enhanced the proliferation ability of cancer cells in c-MYC WT cancer cells, but not in c-MYC S452A cancer cells (Figure 6F-H). These findings confirm that c-MYC S452 phosphorylation is essential for STK16-mediated cancer cell proliferation.

**Targeting STK16 was an effective therapeutic strategy for colorectal cancer treatment**

Based on the above findings, we investigated whether STK16 could be an effective therapeutic target for colorectal cancer. To test our hypothesis, we conducted animal experiments using Lovo cancer cells stably expressing sgcontrol, sgSTK16#1, and sgSTK16#2. Tumor long and short diameters were measured at indicated times, and after 16 days, mice were sacrificed, and tumors were separated, weighed, and imaged. The results demonstrated that loss of STK16 significantly inhibited the growth rate of cancer cells (Figure 7A-C). Additionally, overall survival time was recorded for the three groups of mice, revealing that STK16 knock-out noticeably prolonged the overall survival time (Figure 7D). Immunohistochemistry and western blot assays were performed to assess the expression levels of c-MYC and Ki-67 (representing the proliferation ability of cancer cells), showing that loss of STK16 significantly restrained the expression levels of c-MYC and Ki-67 in vivo (Figure 7E-F). However, achieving STK16 knockout in clinical treatment in vivo is challenging. Therefore, a pharmacological inhibitor might be an effective and achievable approach for clinical application. Subsequently, we applied the STK16 inhibitor (STK16-IN-1) in xenograft experiments. The results indicated that STK16-IN-1 application not only significantly inhibited the growth and weight of the tumor but also prolonged the overall survival time of mice (Figure 7G-J). Consistently, results from immunohistochemistry and western blot assays revealed that STK16-IN-1 significantly decreased the expression levels of c-MYC and Ki-67 (Figure 7K-L). In conclusion, our findings suggest that pharmacologically inhibiting STK16 might be an effective therapeutic approach for colorectal cancer.

**Discussion**

In approximately 70% of human cancers, MYC was believed to be dysregulated, and there was compelling evidence linking aberrant MYC expression to both the initiation and maintenance of tumors.(21) This positioned MYC as one of the most attractive targets for the development of cancer therapeutics. However, lack of typical enzyme active sites made directly inhibiting its function exceptionally challenging.(22) The MYC-MAX heterodimer was essential for MYC binding to DNA and the regulation of gene expression. Hence, some research focused on disrupting the formation of the MYC-MAX heterodimer to block the oncogenic effects of MYC using compounds such as omomyc, saJM589, and KI-MS2-008.(13) However, MYC exerted some of its pro-tumorigenic functions independently of the MYC-MAX heterodimer, making the aforementioned approach less than ideal as a therapeutic strategy. Hence, targeted intervention to modulate c-MYC expression might be an effective therapeutic approach for cancer patients.(22, 23) Post-translational modifications (PTMs) of proteins could impact their activity, expression, subcellular localization, and protein-protein interactions, suggesting targeting the PTMs of c-MYC could be a strategy to influence its expression.(23) Previous studies indicated that c-MYC was subject to various post-translational modifications. CAMKIIγ stabilized the c-Myc protein by directly phosphorylating c-MYC at S62; c-MYC T48 phosphorylation promoted recognition by the E3 ubiquitin ligase component FBW7, destabilizing the c-MYC protein; E3 ubiquitin ligases, such as HUWE1, USP7, USP28, USP7, USP37 and RNF115 played a role in the degradation of c-MYC.(13, 15, 16, 18, 24, 25) In our study, we discovered a new phosphorylation site on c-MYC at S452. We demonstrated that phosphorylation at this site hindered its polyubiquitination. Through in vitro assays, we also found that the absence of c-MYC S452 phosphorylation markedly inhibited the proliferation ability of cancer cells. Also, we found that STK16 was a kinase responsible for catalyzing the phosphorylation of c-MYC at the S452 site. These findings suggest that targeting c-MYC S452 phosphorylation could be a potential therapeutic strategy for colorectal cancer.

Previous research on the biological function of STK16 was limited. Only two studies had revealed that STK16 positively regulated the proliferation ability of lung cancer and fibrosarcoma, with the detailed mechanism by which STK16 regulates tumor growth not thoroughly explored.(11, 12) In the present study, we first revealed the oncogenic role of STK16 in colorectal cancer. We confirmed that STK16 positively regulated the proliferation, migration, and invasion abilities of colorectal cancer cells. Additionally, we provided a detailed explanation of the intrinsic mechanisms by which STK16 mediated colorectal cancer progression. We demonstrated that STK16 promoted colorectal cancer progression by phosphorylating c-MYC at S452, leading to an upregulation of c-MYC expression. Furthermore, we revealed that STK16 promoted colorectal cancer progression dependent on c-MYC S452 phosphorylation. We also confirmed that the STK16 inhibitor significantly suppressed c-MYC expression and the proliferation ability of cancer cells in both in vitro and in vivo experiments.

**Conclusion**

We first revealed the oncogenic function of STK16 in colorectal cancer. Mechanistically, we proved that STK16 phosphorylated c-MYC at serine 452 (S452), which was responsible for STK16-mediated colorectal cancer proliferation. Clinically, we found that loss of STK16 or pharmacological STK16 inhibitor (STK16-IN-1) demonstrated promising effectiveness in inhibiting CRC proliferation by impairing c-MYC oncogene signaling (Figure 7M). These findings elucidated the biological role of STK16 in colorectal cancer and provided new avenues for targeted intervention in the c-MYC signaling pathway.

**List of abbreviations**

c-MYC: MYC proto-oncogene; CDK4: Cyclin dependent kinase 4; CRC: Colorectal cancer; CCK8: Cell counting kit 8; DMEM: Dulbecco's Modified Eagle Medium; GLUT1: Glucose transporter 1; STK16: Serine/threonine kinase 16; TGN: Trans-Golgi Network

**Declarations**

**Ethics approval and consent to participate**

The studies involving human participants were reviewed and approved by the Ethical Committee of People’s Hospital of Xinjiang Uygur Autonomous Region. Participants provided written informed consent to participate. The animal study received approval from the Institutional Animal Care and Use Committee at People’s Hospital of Xinjiang Uygur Autonomous Region.

**Consent for publication**

All the authors listed have approved the manuscript for publication

**Availability of data and material**

All the data of this article were available

**Competing interests**

There was no conflict of interest

**Fundings**

National Natural Science Foundation of Xinjiang Uygur Autonomous Region(2021D01C184); Health Young Medical Talents Special Project of Xinjiang Uygur Autonomous Region (WJWY-202122).

**Authors' contributions**

PL: Overall research design, experimental execution, data analysis and interpretation, initial drafting of the paper. GL: Assisted in experimental procedures, data analysis; BL: Assisted in research design, data analysis and interpretation; TL: Provided research guidance, academic support, and contributed to paper revisions and polishing.

**Acknowledgements**

Not applicable

**Authors' information**

Li Peng E-mail: [lp20014@163.com](mailto:lp20014@163.com)

Liu Guangshi E-mail: [lgs900629@126.com](mailto:lgs900629@126.com)

Lai Bijiang· Wusman E-mail: [lbj437@sina.com](mailto:lbj437@sina.com)

Corresponding Authors: Li Tao; Corresponding E-mail: [lgs900629@126.com](mailto:lgs900629@126.com)

Author affiliation: Gastrointestinal Surgery department, People’s Hospital of Xinjiang Uygur Autonomous Region

**Reference**

1. Bray F, Ferlay J, Soerjomataram I, Siegel RL, Torre LA, Jemal A. Global cancer statistics 2018: GLOBOCAN estimates of incidence and mortality worldwide for 36 cancers in 185 countries. CA Cancer J Clin. 2018;68(6):394-424.

2. Torre LA, Bray F, Siegel RL, Ferlay J, Lortet-Tieulent J, Jemal A. Global cancer statistics, 2012. CA Cancer J Clin. 2015;65(2):87-108.

3. Murray CJL, Lopez AD. Mortality by cause for eight regions of the world: Global Burden of Disease Study. The Lancet. 1997;349(9061):1269-76.

4. Dekker E, Tanis PJ, Vleugels JLA, Kasi PM, Wallace MB. Colorectal cancer. Lancet. 2019;394(10207):1467-80.

5. Wang J, Ji X, Liu J, Zhang X. Serine/Threonine Protein Kinase STK16. Int J Mol Sci. 2019;20(7).

6. Ligos JM, de Lera TL, Hinderlich S, Guinea B, Sanchez L, Roca R, et al. Functional interaction between the Ser/Thr kinase PKL12 and N-acetylglucosamine kinase, a prominent enzyme implicated in the salvage pathway for GlcNAc recycling. J Biol Chem. 2002;277(8):6333-43.

7. Manandhar SP, Siddiqah IM, Cocca SM, Gharakhanian E. A kinase cascade on the yeast lysosomal vacuole regulates its membrane dynamics: conserved kinase Env7 is phosphorylated by casein kinase Yck3. J Biol Chem. 2020;295(34):12262-78.

8. Guinea B, Ligos JM, Lain de Lera T, Martin-Caballero J, Flores J, Gonzalez de la Pena M, et al. Nucleocytoplasmic shuttling of STK16 (PKL12), a Golgi-resident serine/threonine kinase involved in VEGF expression regulation. Exp Cell Res. 2006;312(2):135-44.

9. Lopez-Coral A, Striz AC, Tuma PL. A Serine/Threonine Kinase 16-Based Phospho-Proteomics Screen Identifies WD Repeat Protein-1 As A Regulator Of Constitutive Secretion. Sci Rep. 2018;8(1):13049.

10. Liu J, Yang X, Li B, Wang J, Wang W, Liu J, et al. STK16 regulates actin dynamics to control Golgi organization and cell cycle. Sci Rep. 2017;7:44607.

11. Wang X, Huang R, Lu Z, Wang Z, Chen X, Huang D. Exosomes from M1-polarized macrophages promote apoptosis in lung adenocarcinoma via the miR-181a-5p/ETS1/STK16 axis. Cancer Sci. 2022;113(3):986-1001.

12. In JG, Striz AC, Bernad A, Tuma PL. Serine/threonine kinase 16 and MAL2 regulate constitutive secretion of soluble cargo in hepatic cells. Biochem J. 2014;463(2):201-13.

13. Dang CV. MYC on the path to cancer. Cell. 2012;149(1):22-35.

14. Stine ZE, Walton ZE, Altman BJ, Hsieh AL, Dang CV. MYC, Metabolism, and Cancer. Cancer Discov. 2015;5(10):1024-39.

15. Dang CV, Le A, Gao P. MYC-induced cancer cell energy metabolism and therapeutic opportunities. Clin Cancer Res. 2009;15(21):6479-83.

16. Amati B, Sanchez-Arevalo Lobo VJ. MYC degradation: deubiquitinating enzymes enter the dance. Nat Cell Biol. 2007;9(7):729-31.

17. Duffy MJ, O'Grady S, Tang M, Crown J. MYC as a target for cancer treatment. Cancer Treat Rev. 2021;94:102154.

18. Harrington CT, Sotillo E, Dang CV, Thomas-Tikhonenko A. Tilting MYC toward cancer cell death. Trends Cancer. 2021;7(11):982-94.

19. Wang J, Liu J, Ji X, Zhang X. Tyr198 is the Essential Autophosphorylation Site for STK16 Localization and Kinase Activity. Int J Mol Sci. 2019;20(19).

20. Liu F, Wang J, Yang X, Li B, Wu H, Qi S, et al. Discovery of a Highly Selective STK16 Kinase Inhibitor. ACS Chem Biol. 2016;11(6):1537-43.

21. Meyer N, Penn LZ. Reflecting on 25 years with MYC. Nat Rev Cancer. 2008;8(12):976-90.

22. Llombart V, Mansour MR. Therapeutic targeting of "undruggable" MYC. EBioMedicine. 2022;75:103756.

23. Shim H, Dolde C, Lewis BC, Wu CS, Dang G, Jungmann RA, et al. c-Myc transactivation of LDH-A: implications for tumor metabolism and growth. Proc Natl Acad Sci U S A. 1997;94(13):6658-63.

24. Gu Y, Zhang J, Ma X, Kim BW, Wang H, Li J, et al. Stabilization of the c-Myc Protein by CAMKIIgamma Promotes T Cell Lymphoma. Cancer Cell. 2017;32(1):115-28 e7.

25. Farrell AS, Sears RC. MYC degradation. Cold Spring Harb Perspect Med. 2014;4(3).

**Figure legend**

**Figure 1. STK16 Upregulation in Colorectal Cancer**. **A, B**. Investigation of STK16 expression levels in colorectal cancer using TCGA database. **C**. Representative IHC images of colorectal cancer tissues and adjacent matched normal tissues. **D, E**. Statistical analysis of STK16 expression levels in colorectal cancer tissues and adjacent matched normal tissues. **F**. Immunoblotting (IB) assays to assess STK16 expression in colorectal cancer tissues and adjacent matched normal tissues. **G**. Representative IHC images of colorectal cancer tissues. **H, I**. Statistical analysis of STK16 expression levels in colorectal cancer patients grouped by T stages (**H**) or clinical stages (**I**). **J**. Kaplan-Meier plotter to analyze overall survival time of colorectal cancer patients grouped by STK16 expression. **K**. Kaplan-Meier Plot drawn using TCGA database, with patients grouped by STK16 expression. All IB assays were conducted three times, and consistent results were obtained. Statistical analysis was performed using Student's t-test.

**Figure 2. STK16 Overexpression Promoted Colorectal Cancer Progress In Vitro**. **A**. IB assays validated the successful construction of identified cells. **B-D**. CCK8 (**B**) and Brdu (**C, D**) assays assessed the proliferation ability of colorectal cancer cells stably expressing vector or STK16. **E, F**. Colony formation assays assessed the colony formation ability of colorectal cancer cells stably expressing vector or STK16; Representative images (**E**) and statistical analysis (**F**) shown. **G, H**. Transwell assays assessed the migration and invasion ability of colorectal cancer cells stably expressing vector or STK16; Representative images (**G**) and statistical analysis (**H**) shown. All IB assays were conducted three times, and consistent results were obtained. Statistical analysis used Student's t-test.

**Figure 3. Loss of STK16 Significantly Suppressed Colorectal Cancer Progress In Vitro**. **A**. IB assays validated the successful construction of identified cells. B-D. CCK8 (B) and Brdu (**C, D**) assays assessed the proliferation ability of colorectal cancer cells stably expressing sgCtrl or sgSTK16. **E, F**. Colony formation assays assessed the colony formation ability of colorectal cancer cells stably expressing sgCtrl or sgSTK16; Representative images (**E**) and statistical analysis (**F**) shown. **G, H**. Transwell assays assessed the migration and invasion ability of colorectal cancer cells stably expressing sgCtrl or sgSTK16; Representative images (**G**) and statistical analysis (**H**) shown. All IB assays were conducted three times, and consistent results were obtained. Statistical analysis used Student's t-test.

**Figure 4. STK16 Positively Activated c-MYC Signaling via Stabilizing c-MYC**. **A**. GSEA analysis of TCGA database, patients grouped by the expression level of STK16. **B**. IB assays assessed the expression of c-MYC signaling-related proteins in cancer cells stably expressing sgCtrl or sgSTK16. **C**. Treated cancer cells with autophagy-lysosome pathway inhibitor (chloroquine, CQ) or ubiquitin-proteasome pathway inhibitor (MG132), and immunoblotting assays assessed the expression of c-MYC in HEK293T cells. **D**. Transfected his-ubiquitin plasmid into HEK 293T cells, and IB assays assessed the poly-ubiquitination level of c-MYC in HEK293T cells stably expressing vector or STK16. **E, F**. Transfected his-ubiquitin plasmid into cells, and IB assays assessed the poly-ubiquitination level of c-MYC in HEK293T or RKO cells stably expressing sgCtrl or sgSTK16. **G, H**. CHX was applied to inhibit endogenous protein synthesis, and IB assays assessed the degradation rate of c-MYC in HEK293T cells ectopically expressing vector, STK16 WT, or STK16 T198A. **I**. 63 colorectal cancer samples were collected from People’s Hospital of Xinjiang Uygur Autonomous Region. Statistical analysis of the correlation between the expression level of STK16 and the expression level of c-MYC. All IB assays were conducted three times, and consistent results were obtained. Statistical analysis used Student's t-test.

**Figure 5. STK16 Phosphorylated c-MYC at Serine 452**. **A**. IB and immunoprecipitation (IP) assays confirmed the binding of STK16 and c-MYC. **B**. IB and IP assays assessed the phosphorylation level of c-MYC in RKO cells stably expressing vector, STK16 WT, or STK16 H198A. **C**. IB and IP assays assessed the phosphorylation level of c-MYC in RKO cells stably expressing sgCtrl or sgSTK16. **D, E**. IB and IP assays investigated the binding domain of STK16 and c-MYC. **F**. Transfected c-MYC S430A or c-MYC S452A mutant plasmid into HEK293T cells, and IB and IP assays assessed the phosphorylation level of c-MYC in HEK293T cells stably expressing vector or STK16. **G**. IB and IP assays assessed the poly-ubiquitination level of c-MYC WT, c-MYC S452A, or c-MYC S452E. **H**. IB assays assessed the expression of c-MYC signaling-related proteins in cancer cells stably expressing c-MYC WT, c-MYC S452A, or c-MYC S452E. **I-K**. CCK8 (**I**) and Brdu (**J, K**) assays assessed the proliferation ability of cancer cells stably expressing c-MYC WT, c-MYC S452A, or c-MYC S452E. All IB assays were conducted three times, and consistent results were obtained. Statistical analysis used Student's t-test.

**Figure 6. c-MYC S452 Phosphorylation Was Essential for STK16-Mediated Cancer Cell Proliferation**. **A**. IB assays detected the expression level of identified proteins in colorectal cancer cells stably expressing vector+shnc, STK16+shnc, STK16+shc-MYC#1, or STK16+shc-MYC#2. **B-D**. CCK8 (**B**) and Brdu assays (**C, D**) assessed the proliferation ability of identified cancer cells. **E**. IB assays detected the expression level of identified proteins in colorectal cancer cells stably expressing vector+c-MYC WT, STK16+c-MYC WT, vector+c-MYC S452A, or STK16+c-MYC S452A. **F-H**. CCK8 (**F**) and Brdu assays (**G, H**) assessed the proliferation ability of identified cancer cells. All IB assays were conducted three times, and consistent results were obtained. Statistical analysis used Student's t-test.

**Figure 7. Targeting STK16 Inhibited Colorectal Cancer Proliferation In Vivo**. **A**. Representative tumor images shown. **B**. Drawing tumor growth curve of the three groups (n = 5). **C**. Statistical analysis of tumor weight. **D**. Kaplan-Meier Plot was drawn for the three groups (n = 12). **E, F**. IHC (**E**) and IB (**F**) assays to detect the expression level of identified proteins. **G**. Representative tumor images shown. **H**. Drawing tumor growth curve of the two groups (n = 5). **I**. Statistical analysis of tumor weight. **J**. Kaplan-Meier Plot was drawn for the two groups (n = 13 for DMSO and n = 12 for STK16-IN-1). **K, L**. IHC (**K**) and IB (**L**) assays to detect the expression level of identified proteins. **M**. The working model: STK16 inhibits the ubiquitin-proteasome pathway degradation of c-MYC by phosphorylating c-MYC at S452, contributing to colorectal cancer progress.
