# Supplementary material for: STK16 promoted colorectal cancer progress in a c-MYC signaling-dependent manner
Source: Mol Med. 2024 Apr 15;30:50. doi: 10.1186/s10020-024-00816-9 (PMC11020453; doi:10.1186/s10020-024-00816-9)
Supplement: Supplementary file 4 — Supplementary Material 4 [file 10020_2024_816_MOESM4_ESM.docx]

Corresponding author:

**Department:** Gastrointestinal Surgery department, People’s Hospital of Xinjiang Uygur Autonomous Region

**Academic title:** professor

**Position:** professor
**Name:** Li Tao
**E-mail:** lgs900629@126.com

**Dear Rosamaria Ruggieri**:

We would like to revised the enclosed manuscript entitled “**STK16 promoted colorectal cancer progress in a c-MYC signaling-dependent manner** (MOME-D-23-01616)”. Thank you for your advises, and we have revised your manuscript to improve the manuscript. Main changes are marked mark-manscript draft and answer to editor and reviser also was uploaded in the Point-by-point response and marked manuscript. Other modifications have also been done. No conflict of interest exits in the submission of this manuscript, and manuscript is approved by all authors for publication. I would like to declare on behalf of my co-authors that the work described was original research that has not been published previously, not under consideration for publication elsewhere, in whole or in part, and there is no image duplication, image manipulation, or visual plagiarism. All the authors listed have approved the manuscript that is enclosed. If you have any queries, please don’t hesitate to contact me at the address below.
Thank you and best regards.
Yours sincerely,
