# Supplementary material for: STK16 promoted colorectal cancer progress in a c-MYC signaling-dependent manner
Source: Mol Med. 2024 Apr 15;30:50. doi: 10.1186/s10020-024-00816-9 (PMC11020453; doi:10.1186/s10020-024-00816-9)
Supplement: Supplementary file 7 — Supplementary Material 7 [file 10020_2024_816_MOESM7_ESM.docx]

Dear Rosamaria Ruggieri and reviewers:

We would like to revise the enclosed manuscript entitled “**STK16 promoted colorectal cancer progress in a c-MYC signaling-dependent manner** (MOME-D-23-01616R1)”. We are grateful for your recognition on our study. We appreciated the reviewers’ comments on our study. We have indeed taken these reviewers’ comments very seriously. New experiments were conducted to address the reviews’ concerns. We have provided point-by-point answers to address all the reviewers’ comments.

**The point-by-point responses to the reviewers’ comments are as followed:**

**Reviewer #1**

**Q1**. In Fig. 4H, it should be sgSTK16#1 and #2. In Fig. 5I & K, it should be S452A and S452E. In Fig. 7B-D, it should be sgSTK16#1 and #2.

**A**: Thank you for your mention. I have changed it in our revised paper.

**Q2**. In Fig. 4I, please describe the tissue source and sample size in the figure legend.

**A**: Thank you for your mention. I have added it in our revised paper as followed: 63 colorectal cancer samples were collected from People’s Hospital of Xinjiang Uygur Autonomous Region.

**Q3**. Please elaborate more on the other phosphorylation sites occurred in c-Myc, such as T58 and S62, and their effect on the stability of c-Myc in the Discussion section.

**A**: Thank you for your mention. I have added it in our revised paper as followed: CAMKIIγ stabilized the c-Myc protein by directly phosphorylating c-MYC at S62; c-MYC T48 phosphorylation promoted recognition by the E3 ubiquitin ligase component FBW7, destabilizing the c-MYC protein.
